# Association between growth factors and Sjögren syndrome: A two-sample Mendelian randomization study

**DOI:** 10.1097/MD.0000000000042210

**Published:** 2025-04-18

**Authors:** Zuoyuan Cao, Zhujing Zhu, Huanru Qu

**Affiliations:** a Department of Rheumatology and Clinical Immunology, Longhua Hospital Shanghai University of Traditional Chinese Medicine, Shanghai, China.

**Keywords:** causal association, epidermal growth factor, growth factor, Mendelian randomization, Sjögren syndrome

## Abstract

Previous observational studies have suggested a link between growth factors and Sjögren syndrome, but a definitive causal relationship has yet to be established. We conducted a two-sample Mendelian randomization (MR) study using summary statistics from the FinnGen study on Sjögren syndrome and various growth factors. Analytical techniques included inverse variance weighted, MR-Egger, weighted median, and weighted mode to explore potential causal links. Our analysis indicated that genetically determined growth factors did not have a causal effect on Sjögren syndrome. The inverse variance weighted method showed nonsignificant associations for various exposures, including epidermal growth factor (odds ratios [OR] = 0.94, *P* = .720), proheparin-binding EGF-like growth factor (OR = 0.95, *P* = .621), VEGF sR2 (OR = 1.0, *P* = .988), FGF7 (OR = 1.08, *P* = .519), PDGF-AA (OR = 0.87, *P* = .218), VEGF121 (OR = 0.93, *P* = .466), TGF-β R II (OR = 1.01, *P* = .934), and NGFI-A-binding protein 2 (OR = 1.13, *P* = .264). The MR-Egger, weighted median, and weighted mode methods supported these findings. Our study did not find significant causal associations between genetic levels of the studied growth factors and the onset of Sjögren syndrome. Future research should investigate more detailed genetic interactions and modifiable environmental factors to better understand pathways relevant to the prevention and management of Sjögren syndrome.

## 1. Introduction

Sjögren syndrome is a chronic autoimmune disorder that predominantly affects women. The disease is characterized by dysfunction of the exocrine glands, such as the parotid and lacrimal glands, leading to symptoms like dry mouth and dry eyes.^[1]^ Fatigue, arthralgia and myalgia are also common symptoms, whereas extraglandular manifestations that involve the respiratory, nervous and vascular systems occur in a subset of patients, and patients with involvement of the pulmonary, renal, hematological, central, and peripheral nervous systems have a poorer prognosis.^[2]^ The condition can be classified into primary Sjögren syndrome, where it occurs independently, and secondary Sjögren syndrome, where it coexists with other connective tissue diseases.^[3]^ Epidemiologically, Sjögren syndrome has an incidence rate of approximately 6.92 per 100,000 person-years and a prevalence rate of 60.82 per 100,000 inhabitants, with a peak onset at 56 years of age.^[4]^ Patients often endure persistent and severe pain, along with multiple physical symptoms including dental caries, vaginal dryness, and arthralgia.^[5]^

Growth factors, a group of bioactive protein agents, play pivotal roles in tissue growth and cell fate regulation. These factors are categorized into various families such as transforming growth factors (TGFs), bone morphogenetic proteins, connective tissue growth factors, vascular endothelial growth factors (VEGFs), and insulin-like growth factors.^[6,7]^ For example, BAFF, a member of the TNF family and a type of growth factor, is crucial for B cell survival and is implicated in enhancing lymphocytic infiltration and autoantibody production in Sjogren syndrome.^[8,9]^

Epidemiological studies have consistently observed associations between elevated levels of certain growth factors and an increased risk or severity of autoimmune conditions, suggesting a potential role for these proteins in the development or exacerbation of Sjögren syndrome. Research has shown varying levels of growth factors like epidermal growth factor (EGF) and TGF-α in patients with Sjogren syndrome, which are linked to clinical manifestations such as severe intraoral complications and laryngopharyngeal reflux.^[10,11]^ However, these findings are not universally consistent, as other studies report no significant differences in growth factors such as VEGF-A between Sjogren patients and healthy controls, and do not establish clear pathogenic pathways.^[12]^ This variability highlights the limitations of observational studies, which often cannot definitively establish causality due to issues like confounding factors and reverse causation.

In this context, Mendelian randomization (MR) emerges as a powerful epidemiological tool. By leveraging genetic variants as proxies for modifiable exposures, MR utilizes the random distribution of alleles during meiosis to provide a methodologically robust approach that mitigates the biases typically encountered in observational research.^[13–15]^ This study applies a two-sample MR analysis, utilizing genome-wide association study (GWAS) summary statistics, to rigorously assess the potential causal relationship between specific growth factors and the onset of Sjögren syndrome. Through this approach, we aim to clarify whether the observed epidemiological links reflect genuine causal relationships or are merely associations confounded by other factors.

## 2. Materials and methods

### 2.1. Study design and data sources

**Figure 1.**
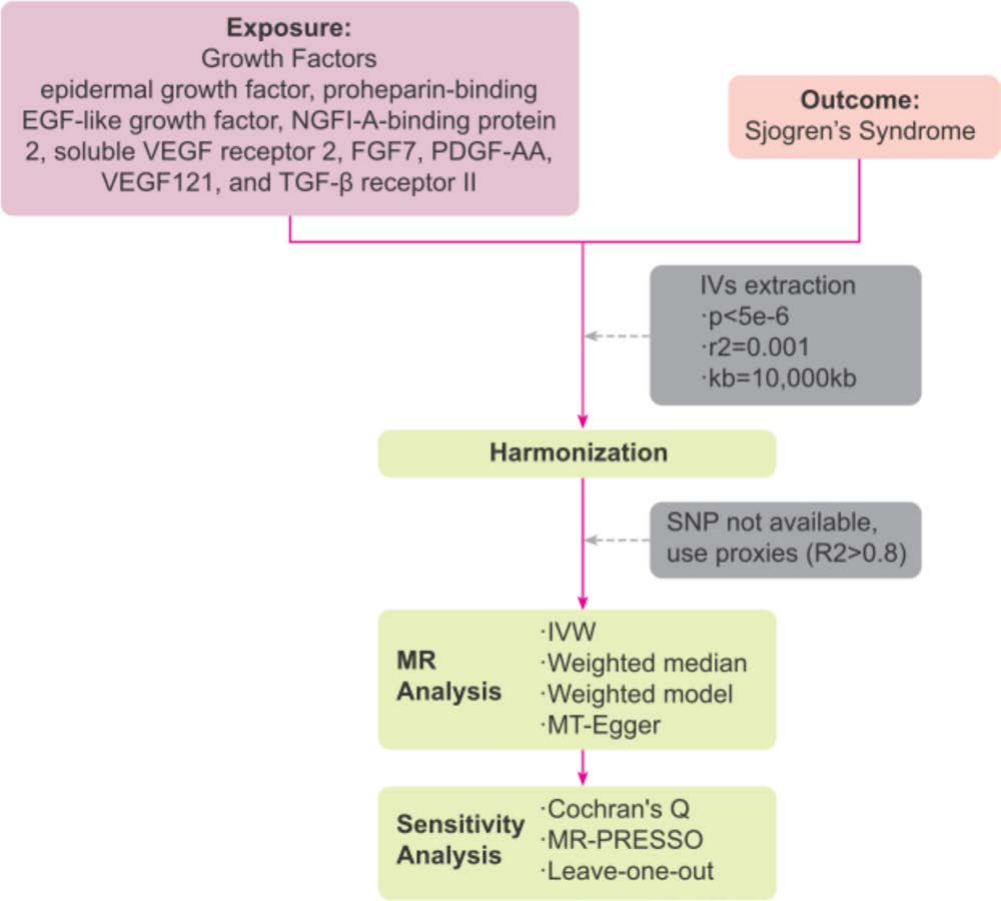
Schematic illustration of two-sample MR analysis.

This study utilized a two-sample MR design. Informed consent and ethical approval were secured from the original studies. The methodology operates under several critical assumptions for the validity of causal estimates in MR studies: (1) Instrumental variables (IVs) must be robustly associated with the exposure; (2) IVs should not be linked with any potential confounding factors; (3) IVs must influence the outcome solely through the exposure pathway.^[16]^ The flowchart of MR analyses in this study is shown in Figure 1.

GWAS summary statistics for Sjögren syndrome and growth factors^[17]^ were acquired from prior research conducted based on FinnGen study. Therefore, additional ethical approval was not required. The FinnGen study (https://www.finngen.fi/en) is a public–private partnership formed in 2017, comprising Finnish universities, biobanks, hospital districts, and multiple pharmaceutical companies. Its objective is to amalgamate National Health Record and genetic data from approximately 500,000 Finnish individuals. Study participants encompass patients with both acute and chronic diseases as well as healthy volunteers and population cohorts. The dataset for Sjögren syndrome includes 1290 cases and 213,145 controls, totaling 16,380,454 single nucleotide polymorphisms (SNPs) (finn-b-M13_SJOGREN). The dataset for growth factors includes levels of epidermal growth factor, proheparin-binding EGF-like growth factor, NGFI-A-binding protein 2, soluble VEGF receptor 2, FGF7, PDGF-AA, VEGF121, and TGF-β receptor II. Detailed information is presented in Table 1.

**Table 1:** Description of GWAS summary samples used in this study.

Trait	GWAS ID	Case/control	SNPs
Sjögren syndrome	finn-b-M13_SJOGREN	1290/213,145	16,380,454
Epidermal growth factor levels	ebi-a-GCST90012001	21,758	20,300,000
Proheparin-binding EGF-like growth factor levels	ebi-a-GCST90010133	1300	13,419,876
NGFI-A-binding protein 2	prot-a-1993	3301	10,534,735
VEGF sR2	prot-c-3651_50_5	–	501,428
FGF7	prot-c-4487_1_1	–	501,428
PDGF-AA	prot-c-4499_21_1	–	501,428
VEGF121	prot-c-4867_15_2	–	501,428
TGF-βRII	prot-c-5133_17_3	–	501,428

GWAS = genome-wide association study, SNP = single nucleotide polymorphism.

### 2.2. Instrumental variable

To ensure the precision and credibility of our conclusions on causal relationships, we implemented a series of stringent quality control measures to select the most appropriate genetic instruments. Initially, we used a set of SNPs that met the genome-wide significance threshold (*P* < 5 × 10^‐6^) as IVs.^[18]^ We then retained SNPs with a minor allele frequency of more than 0.01. To minimize the impact of linkage disequilibrium (LD), SNPs were further filtered based on an LD measure (*R*^2^) of <0.001 and within a genomic region size of 10,000 kb.^[19]^ In cases where the selected SNPs were absent in the summary data of the outcome, proxy SNPs with a high LD correlation (*R*^2^ > 0.8) were selected for substitution.^[20]^ The *F*-statistic, calculated as *R*^2^ × (N ‐ 2)/(1 ‐ *R*^2^) for each IV, served as a metric for instrumental strength, where *R*^2^ represents the proportion of phenotypic variance explained by each genetic variant in the exposure, and N denotes the sample size.^[21]^ Generally, an *F*-statistic exceeding 10 was established as the threshold for robust instrumental variables.^[22]^

### 2.3. Statistical analysis

#### 2.3.1. Main analyses

We performed two-sample MR analyses using the TwoSampleMR package in R (version 4.0.5). The primary analysis method was inverse variance weighted (IVW).^[23]^ Corresponding odds ratios (ORs) and 95% confidence intervals (95% CIs) were calculated to assess the causal association between exposure and the risk of outcomes. MR-Egger regression was used to estimate the causal effect and to test for directional pleiotropy via the MR-Egger intercept test; a nonsignificant intercept (*P* > .05) suggests no pleiotropy.^[24]^ We applied the false discovery rate correction to adjust *P*-values for multiple testing, with significance set at PFDR < .05.

#### 2.3.2. Pleiotropy and sensitivity analysis

To evaluate the presence of horizontal pleiotropy, we utilized several methods. MR-Egger regression allowed us to estimate the average pleiotropic effect, with its intercept term as an indicator.^[24]^ We assessed asymmetry in the funnel plot as another indicator of horizontal pleiotropy.^[25]^ The MR Pleiotropy Residual Sum and Outlier (MR-PRESSO) test was employed to detect, correct for, and assess the impact of horizontal pleiotropy by identifying and removing outliers.^[26]^ Heterogeneity was assessed using the IVW approach and MR-Egger regression, quantified by Cochran *Q* statistic.^[27]^ A leave-one-out analysis was conducted to test the robustness and consistency of our findings.^[28]^

## 3. Results

### 3.1. Selection of instrumental variables

In this MR analysis, various exposures were assessed using different numbers of IVs. Epidermal growth factor levels were analyzed with 14 IVs (average *F*-statistic of 25), while proheparin-binding EGF-like growth factors were analyzed with 5 IVs (average *F*-statistic of 30). NGFI-A-binding protein 2 utilized 19 IVs (average *F*-statistic of 25), VEGF sR2 employed 7 IVs (average *F*-statistic of 38), and the exposures FGF7, PDGF-AA, VEGF121, and TGF-βR II involved 3, 3, 4, and 4 IVs, respectively, with average *F*-statistics of 22, 24, 27, and 22. Concerning SNP compatibility, and for epidermal growth factor levels, 1 SNP could not be replaced due to the absence of a suitable proxy. Detailed information regarding the number of IVs and proxy relationships for each exposure is summarized in Table S1, Supplemental Digital Content, http://links.lww.com/MD/O688.

### 3.2. Causal effects of growth factors on Sjögren syndrome

**Figure 2.**
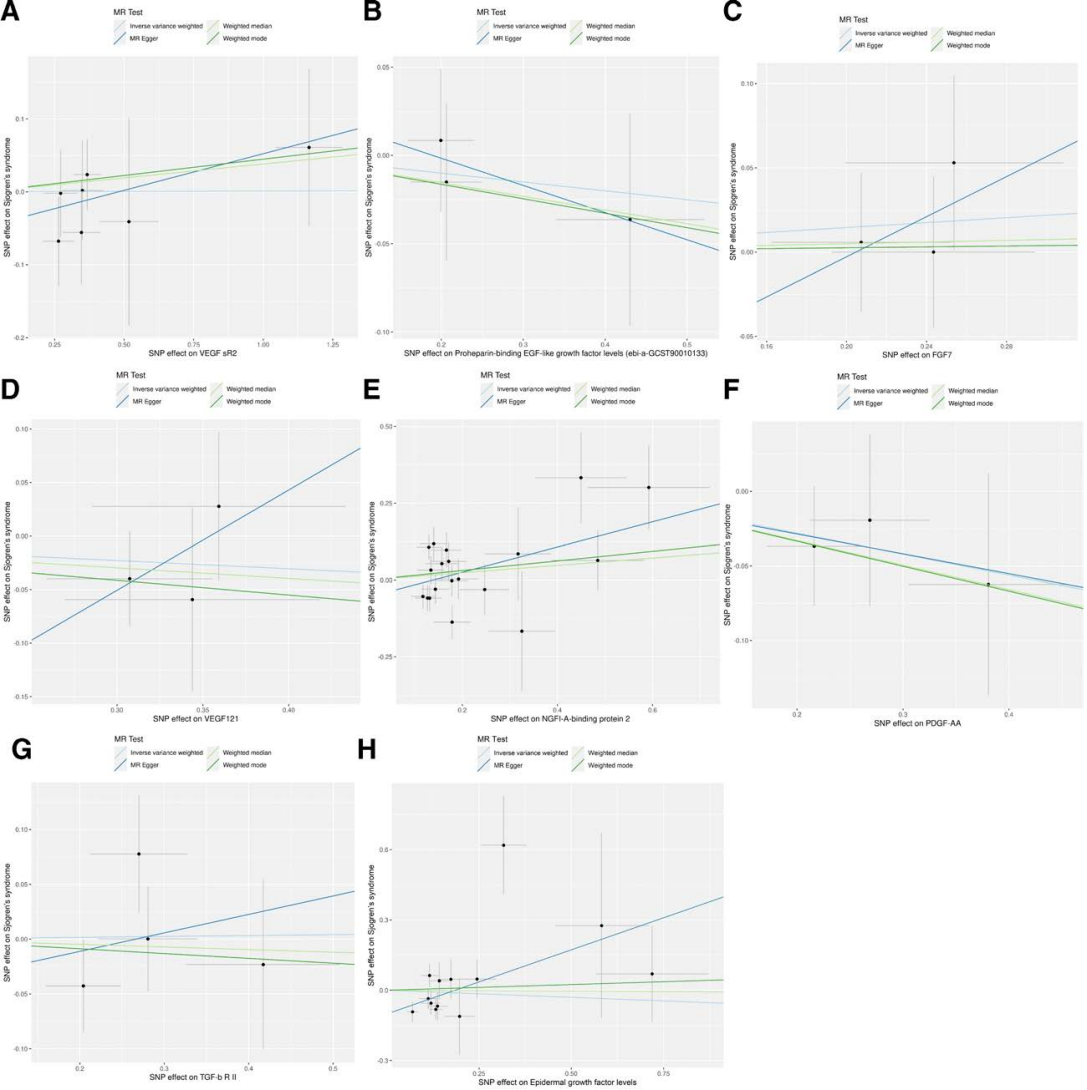
Scatter plots of the causal relationships between growth factors and Sjögren syndrome. (A) Causal effect of VEGF sR2 on Sjögren syndrome. (B) Causal effect of proheparin-binding EGF-like growth factor levels on Sjögren syndrome. (C) Causal effect of FGF7 on Sjögren syndrome. (D) Causal effect of VEGF121 on Sjögren syndrome. (E) Causal effect of NGFI-A-binding protein 2 on Sjögren syndrome. (F) Causal effect of PDGF-AA on Sjögren syndrome. (G) TGF-β R II on Sjögren syndrome. (H) Epidermal growth factor levels on Sjögren syndrome.The slope of each line corresponding to the causal estimates for each method. Individual SNP-effect on the outcome (point and vertical line) against its effect on the exposure (point and horizontal line) was delineated in the background.

Overall, there were no causal associations between the various exposures studied and Sjögren syndrome (Fig. 2), and the MR estimates of different methods were presented in Table 2. As indicated by the IVW method results: VEGF sR2 reported an OR of 1.00 (95% CI: 0.89–1.13, *P* = .988); proheparin-binding EGF-like growth factor showed an OR of 0.95 (95% CI: 0.78–1.16, *P* = .621); FGF7 indicated an OR of 1.08 (95% CI: 0.86–1.34, *P* = .519); VEGF121 had an OR of 0.93 (95% CI: 0.76–1.14, *P* = .466); NGFI-A-binding protein 2 had an OR of 1.13 (95% CI: 0.91–1.39, *P* = .264); PDGF-AA showed an OR of 0.87 (95% CI: 0.7–1.09, *P* = .218); TGF-β R II reported an OR of 1.01 (95% CI: 0.83–1.22, *P* = .934); and epidermal growth factor levels had an OR of 0.94 (95% CI: 0.68–1.3, *P* = .720). All other methods, including MR-Egger, the weighted median, and the weighted mode, showed consistent results, affirming the absence of significant associations.

**Table 2:** MR estimates for the causal effect.

Exposure	Outcome	Methods	SNP	*P* value	OR (95% CI)
Epidermal growth factor levels	Sjögren syndrome	Inverse variance weighted	13	.720	0.94 (0.68–1.3)
		MR-Egger	13	.110	1.73 (0.94–3.19)
		Weighted median	13	.970	0.99 (0.71–1.39)
		Weighted mode	13	.840	1.05 (0.65–1.69)
Proheparin-binding EGF-like growth factor levels	Sjögren syndrome	Inverse variance weighted	3	.621	0.95 (0.78–1.16)
		MR-Egger	3	.693	0.86 (0.48–1.53)
		Weighted median	3	.515	0.93 (0.73–1.17)
		Weighted mode	3	.620	0.92 (0.7–1.21)
NGFI-A-binding protein 2	Sjögren syndrome	Inverse variance weighted	19	.264	1.13 (0.91–1.39)
		MR-Egger	19	.125	1.51 (0.92–2.48)
		Weighted median	19	.343	1.13 (0.88–1.44)
		Weighted mode	19	.443	1.17 (0.79–1.72)
VEGFsR2	Sjögren syndrome	Inverse variance weighted	7	.988	1 (0.89–1.13)
		MR-Egger	7	.466	1.11 (0.86–1.42)
		Weighted median	7	.602	1.04 (0.9–1.2)
		Weighted mode	7	.589	1.05 (0.9–1.22)
FGF7	Sjögren syndrome	Inverse variance weighted	3	.519	1.08 (0.86–1.34)
		MR-Egger	3	.725	1.81 (0.14–22.77)
		Weighted median	3	.862	1.03 (0.78–1.35)
		Weighted mode	3	.943	1.01 (0.74–1.38)
PDGF-AA	Sjögren syndrome	Inverse variance weighted	3	.218	0.87 (0.7–1.09)
		MR-Egger	3	.838	0.88 (0.32–2.38)
		Weighted median	3	.211	0.85 (0.66–1.1)
		Weighted mode	3	.395	0.85 (0.62–1.15)
VEGF121	Sjögren syndrome	Inverse variance weighted	3	.466	0.93 (0.76–1.14)
		MR-Egger	3	.642	2.55 (0.14–46.81)
		Weighted median	3	.399	0.91 (0.72–1.14)
		Weighted mode	3	.423	0.87 (0.67–1.14)
TGF-βRII	Sjögren syndrome	Inverse variance weighted	4	.934	1.01 (0.83–1.22)
		MR-Egger	4	.770	1.18 (0.44–3.18)
		Weighted median	4	.840	0.98 (0.77–1.23)
		Weighted mode	4	.797	0.96 (0.7–1.3)

CI = confidence interval, OR = odds ratio, SNP = single nucleotide polymorphism.

The forest plot for effect sizes of SNPs for growth factors and those for Sjögren syndrome were shown in Figure S1, Supplemental Digital Content, http://links.lww.com/MD/O689. Significant heterogeneity was observed for epidermal growth factor levels (*Q* statistic = 23.37, *P* = .02) and NGFI-A-binding protein 2 (*Q* statistic = 38.33, *P* = .004), suggesting variability in causal estimates. For these variables, the MR-PRESSO test also indicated significant global distortion, with 1 outlier identified for each and corrected outcomes showing OR = 1.02 for epidermal growth factor levels and OR = 1.19 for NGFI-A-binding protein 2 (Tables S2 and S3, Supplemental Digital Content, http://links.lww.com/MD/O690). Horizontal pleiotropy was generally unlikely to affect the causality assessments, as indicated by nonsignificant MR-Egger intercepts for all exposures. Other exposures demonstrated neither significant heterogeneity nor pleiotropy. Leave-one-out analysis indicated that the causal estimates of growth factors were not driven by any single SNP. The funnel plots and leave-one-out analysis plots were shown in Figures S2 and S3, Supplemental Digital Content, http://links.lww.com/MD/O689.

## 4. Discussion

This groundbreaking study embarked on investigating the causal relationship between growth factors and Sjögren syndrome using a comprehensive array of MR techniques. Despite the extensive analysis across multiple MR methods, our study did not find evidence supporting a causal link between genetic predispositions to altered growth factor levels and the risk of developing Sjögren syndrome. This result prompts further exploration into the nuanced relationships between genetic variants influencing growth factors and the pathogenesis of Sjögren syndrome.

In the broader context of our MR findings, which did not establish a causal relationship between growth factors and Sjogren syndrome, the existing literature presents mixed results. One study found significantly lower salivary EGF levels in Sjögren syndrome patients compared to controls, linking these lower levels to more severe intraoral manifestations.^[10]^ Similarly, research on patients with Sjogren syndrome and laryngopharyngeal reflux observed elevated levels of salivary transforming growth factor alpha compared to healthy controls, potentially indicating an association with the severity of reflux symptoms.^[11]^ Another study, however, found no significant differences in EGF levels between patients with Sjögren syndrome and controls, despite the prevalence of reflux conditions in these patients.^[29]^ Additionally, another research found no statistically significant differences in VEGF-A levels between primary Sjogren syndrome patients and healthy controls, although VEGF-A correlated with several inflammation markers.^[12]^ Similarly, a study comparing VEGF and EGF levels in Sjögren syndrome patients and healthy subjects reported that the balance of VEGF isoforms was not disturbed, suggesting these growth factors might not serve as biomarkers for the disease.^[30]^ Finally, a comprehensive study on immune cell alterations in primary Sjögren syndrome highlighted the complex immune regulation in the disease but did not link these changes directly to growth factor levels, instead focusing on broader signaling pathways and gene expression changes.^[31]^ These findings highlight the complex role of growth factors in Sjögren syndrome, suggesting that while they may contribute to disease manifestations or inflammation, they are not causal factors in disease onset.

Building on the established epidemiological evidence, there are several plausible explanations for the observed lack of association in our study between growth factors and Sjögren syndrome. While studies have demonstrated the involvement of growth factors like TGF-β1 in the immune-mediated inflammation and fibrosis observed in Sjogren syndrome, indicating their role in the disease’s progression rather than its initiation,^[32,33]^ other research suggests that these factors may not trigger the initial disease onset. For instance, neurotrophins, which have been implicated in the activation of immune cells in Sjögren syndrome, show a direct correlation with disease activity, yet their initial causative role remains uncertain.^[34]^ Additionally, the genetic variants used as instrumental variables in our study might not capture all the biological pathways that mediate the effects of growth factors on Sjögren syndrome. For example, EGF has been shown to interact with signaling pathways like STAT1 and STAT4 in cell culture models of the disease, suggesting complex regulatory mechanisms that our genetic instruments may not fully encompass.^[35]^ Similarly, the role of the TGF-β pathway in salivary gland fibrosis highlights the multifaceted impact of growth factors on tissue pathology, which may not be directly linked to disease causation but rather to progression and severity.^[36]^ These findings underscore the need for broader genetic exploration and more detailed pathway analyses to better understand the multifactorial nature of Sjögren syndrome and the potential therapeutic targets within these complex biological networks.

This study’s major strength lies in its use of MR, which leverages genetic variants as instrumental variables to robustly assess causal relationships, minimizing the confounding and reverse causation typical in observational studies. The comprehensive dataset from the FinnGen study, encompassing a broad array of growth factors, along with the application of multiple MR methods such as IVW, MR-Egger, weighted median, and weighted mode, enhances the reliability of our findings by ensuring consistency across various statistical models.

However, the study is not without limitations. Firstly, the potential for pleiotropic effects where genetic variants might influence the outcome via pathways other than those involving the exposure of interest remains a concern, despite attempts to mitigate this through sensitivity analyses like MR-Egger and MR-PRESSO. Secondly, the generalizability of our findings may be limited due to the study population primarily consisting of individuals of Finnish descent, which might not reflect the genetic and environmental variability present in broader global populations. Additionally, due to the significant heterogeneity in the clinical manifestations of Sjögren syndrome, this study did not further differentiate the growth factor levels in patients with organ-specific involvement, aiming to elucidate the pathogenesis of different clinical subtypes. These factors suggest a need for further research involving more diverse populations and additional genetic markers to fully elucidate the relationships between growth factors and Sjögren syndrome. Lastly, this study did not stratify the analysis by disease duration, primary versus secondary Sjögren syndrome subtypes, or other comorbidities. These factors may indirectly interfere with causal inference by affecting growth factor levels. Future research needs to further refine patient subgroups to explore heterogeneity.

## 5. Conclusions

In conclusion, our research did not establish a direct causal connection between genetically influenced levels of the specific growth factors analyzed and Sjögren syndrome. The findings highlight the necessity for future studies to employ more advanced genetic analytical approaches, incorporate richer GWAS summary data, and use a broader selection of genetic instruments to further investigate and potentially validate these initial findings.

## Author contributions

**Writing – original draft:** Zuoyuan Cao, Zhujing Zhu, Huanru Qu.

**Writing – review & editing:** Zuoyuan Cao, Zhujing Zhu, Huanru Qu.

## Supplementary Material


