# Supplementary material for: Association between growth factors and Sjögren syndrome: A two-sample Mendelian randomization study
Source: Medicine (Baltimore). 2025 Apr 18;104(16):e42210. doi: 10.1097/MD.0000000000042210 (PMC12014104; doi:10.1097/MD.0000000000042210)
Supplement: Supplementary file 1 [file medi-104-e42210-s001.docx]

**Supplemental Digital Content Table1 Summary of Instrumental Variables and Proxy Relationships Across Exposures**

| Exposure | Number of IVs | F-value (Min-Max) | SNP Match and Proxy Information |
| --- | --- | --- | --- |
| Epidermal growth factor levels | 14 | 25 (21-54) | Proxy SNP replacement: None (rs193287776 has no proxy) |
| Proheparin-binding EGF-like growth factor | 5 | 30 (21-43) | - |
| NGFI-A-binding protein 2 | 19 | 25 (22-35) | - |
| VEGF sR2 | 7 | 38 (21-93) | - |
| FGF7 | 3 | 22 (21-23) | - |
| PDGF-AA | 3 | 24 (22-26) | - |
| VEGF121 | 4 | 27 (21-40) | - |
| TGF-b R II | 4 | 22 (21-22) | - |
