# Supplementary material for: Association between growth factors and Sjögren syndrome: A two-sample Mendelian randomization study
Source: Medicine (Baltimore). 2025 Apr 18;104(16):e42210. doi: 10.1097/MD.0000000000042210 (PMC12014104; doi:10.1097/MD.0000000000042210)

**Supplemental Digital Content Figure1**. **Forest plots that estimated the causal associations between Growth Factors and Sjögren’s Syndrome by using two-sample MR analysis.**(A)VEGF sR2 on Sjögren’s syndrome; (B) Proheparin-binding EGF-like growth factor levels on Sjögren’s syndrome; (C) FGF7 on Sjögren’s syndrome; (D) VEGF121 on Sjögren’s syndrome; (E) NGFI-A-binding protein 2 on Sjögren’s syndrome; (F) PDGF-AA on Sjögren’s syndrome; (G) TGF-βR II on Sjögren’s syndrome; (H) Epidermal Growth Factor Levels on Sjögren’s syndrome.


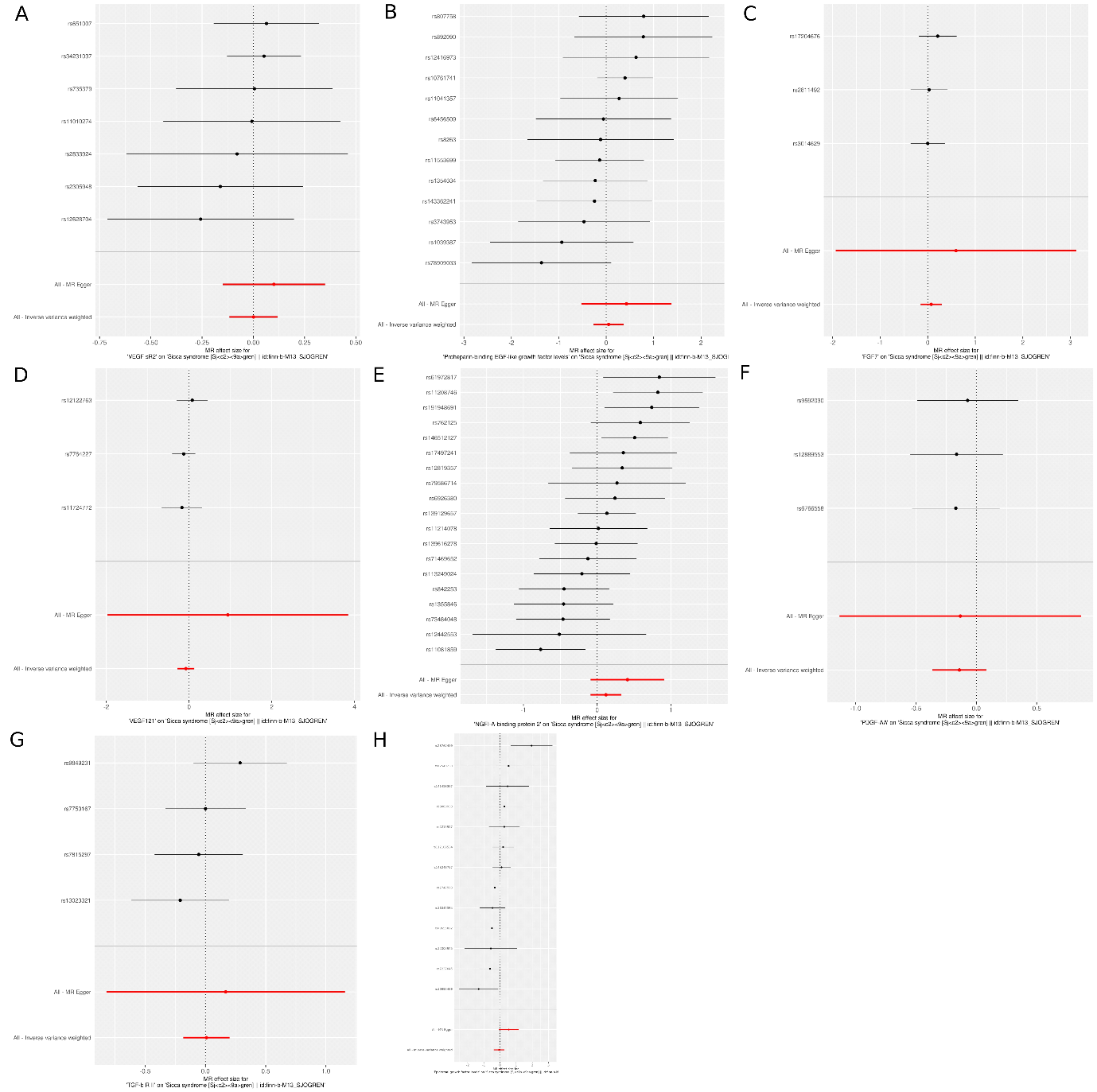


**Supplemental Digital Content Figure2. Funnel plots were applied to detect whether the observed associations between Growth Factors and Sjögren’s Syndrome, were along with obvious heterogeneity.**(A) VEGF sR2; (B) Proheparin-binding EGF-like growth factor; (C) FGF7; (D) VEGF121; (E) NGFI-A-binding protein 2; (F) PDGF-AA; (G) TGF-β R II; (H) Epidermal Growth Factor Levels.


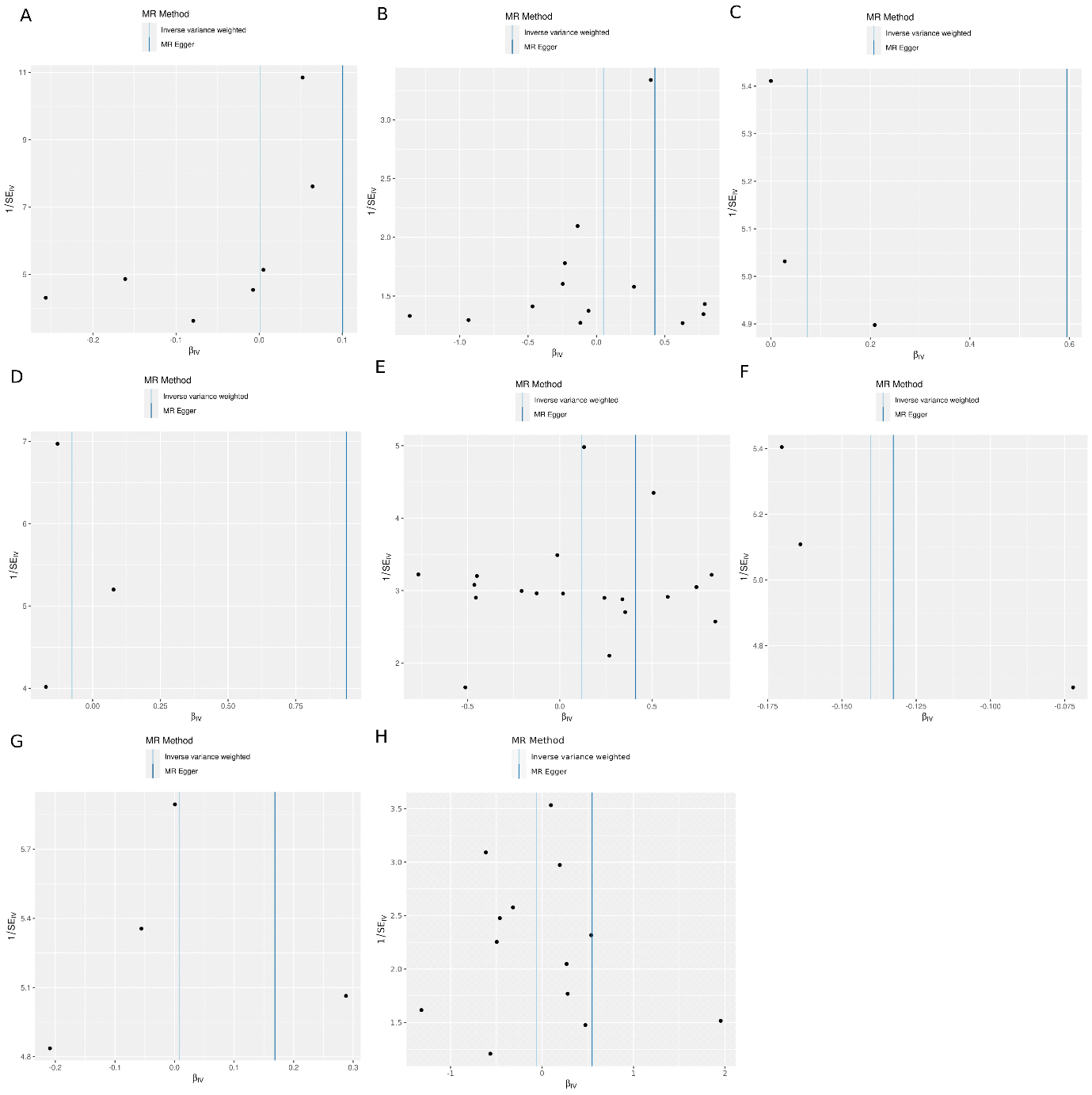


**Supplemental Digital Content Figure3. Leave‐one‐out analyses for the forward MR analysis to evaluate whether any single instrumental variable was driving the causal effect.** (A) VEGF sR2; (B) Proheparin-binding EGF-like growth factor; (C) FGF7; (D) VEGF121; (E) NGFI-A-binding protein 2; (F) PDGF-AA; (G) TGF-β R II; (H) Epidermal Growth Factor Levels.


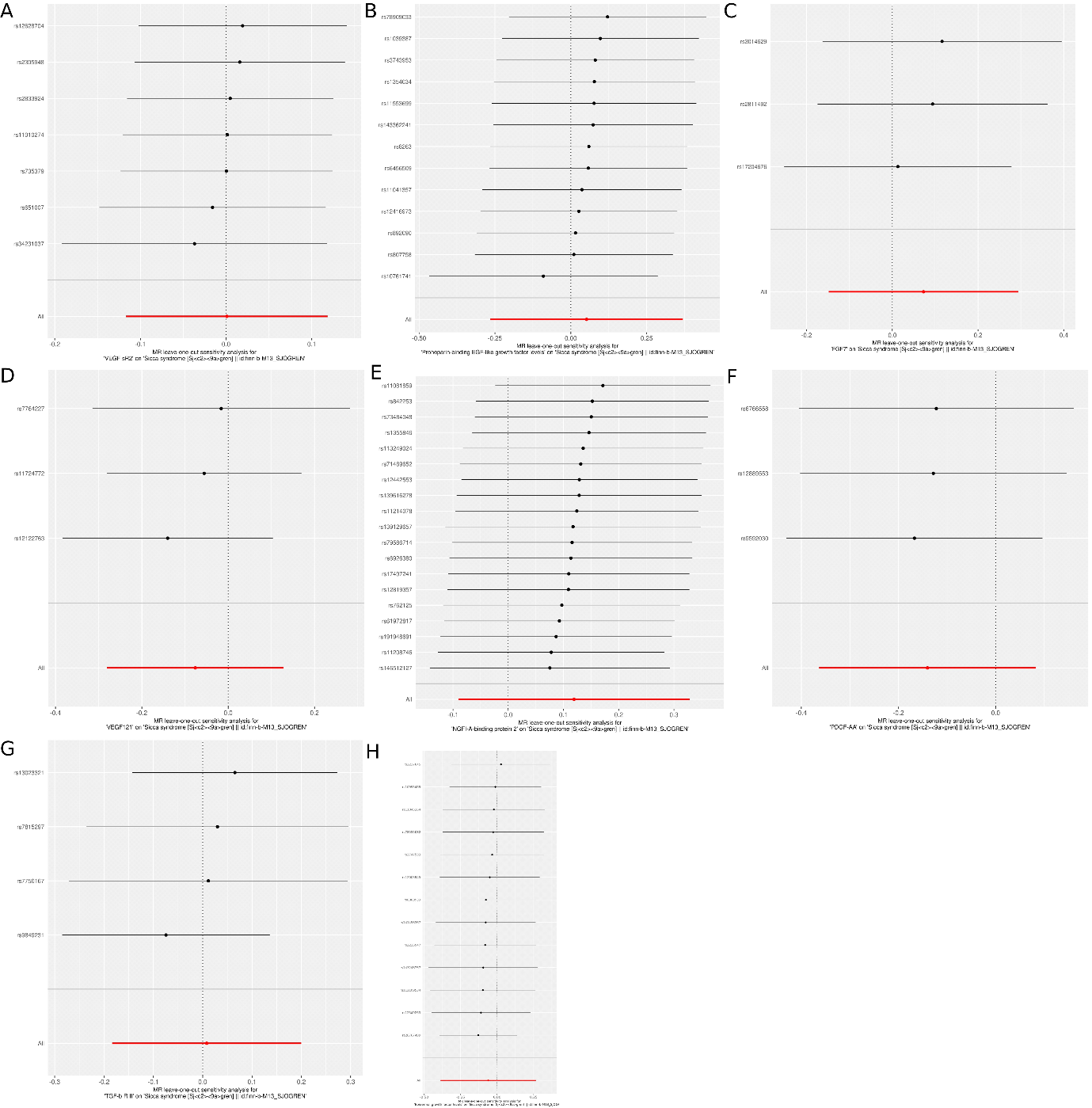

Supplement: Supplementary file 2 [file medi-104-e42210-s002.docx]
