# Supplementary material for: Association between growth factors and Sjögren syndrome: A two-sample Mendelian randomization study
Source: Medicine (Baltimore). 2025 Apr 18;104(16):e42210. doi: 10.1097/MD.0000000000042210 (PMC12014104; doi:10.1097/MD.0000000000042210)
Supplement: Supplementary file 3 [file medi-104-e42210-s003.docx]

**Supplemental Digital Content Table2** **Heterogeneity and pleiotropy**

| Exposure | Outcome | Heterogeneity | |  | Pleiotropy | |
| --- | --- | --- | --- | --- | --- | --- |
|  |  | Q statistic (IVW) | P value |  | MR-Egger Intercept | P value |
| Epidermal growth factor levels | Sjögren’s syndrome | 23.37 | 0.02 |  | -0.10 | 0.05 |
| Proheparin-binding EGF-like growth factor levels |  | 0.282 | 0.869 |  | 0.029 | 0.771 |
| NGFI-A-binding protein 2 |  | 38.330 | 0.004 |  | -0.057 | 0.225 |
| VEGF sR2 |  | 2.483 | 0.870 |  | -0.048 | 0.416 |
| FGF7 |  | 0.650 | 0.722 |  | -0.122 | 0.755 |
| PDGF-AA |  | 0.142 | 0.931 |  | -0.002 | 0.990 |
| VEGF121 |  | 0.923 | 0.630 |  | -0.332 | 0.618 |
| TGF-βRII |  | 3.230 | 0.357 |  | -0.045 | 0.775 |

**Supplemental Digital Content Table3** **MR-PRESSO**

| Exposure | Outcome | RAW | | Outlier corrected | | Global P | Number of outliers | Distortion P |
| --- | --- | --- | --- | --- | --- | --- | --- | --- |
|  |  | OR | P | OR | P |  |  |  |
| Epidermal growth factor levels | Sjögren’s syndrome | 0.94 (0.68 - 1.30) | 0.72 | 1.02(0.94-1.11) | 0.590 | 0.001 | 1  rs4644164 | 0.205 |
| NGFI-A-binding protein 2 |  | 1.13 (0.91 - 1.39) | 0.279 | 1.19 (0.98 - 1.44) | 0.103 | 0.004 | 1  rs11214078 | 0.697 |
| Proheparin-binding EGF-like growth factor levels |  | 1.11 (0.88 - 1.4) | 0.388 | 1.07 (0.93 - 1.22) | 0.360 | <0.001 | 1  rs251762 | 0.294 |
| TGF-β RII |  | 1.01 (0.83 - 1.22) | 0.939 | / | / | / | / | / |
| VEGFsR2 |  | 1 (0.93 - 1.08) | 0.983 | / | / | / | / | / |
| VEGF121 |  | 1.03 (0.82 - 1.3) | 0.832 | / | / | / | / | / |
